# ROS of Distinct Sources and Salicylic Acid Separate Elevated CO_2_-Mediated Stomatal Movements in Arabidopsis

**DOI:** 10.3389/fpls.2020.00542

**Published:** 2020-05-08

**Authors:** Jingjing He, Ruo-Xi Zhang, Dae Sung Kim, Peng Sun, Honggang Liu, Zhongming Liu, Alistair M. Hetherington, Yun-Kuan Liang

**Affiliations:** ^1^State Key Laboratory of Hybrid Rice, Department of Plant Science, College of Life Sciences, Wuhan University, Wuhan, China; ^2^School of Biological Sciences, Life Sciences Building, University of Bristol, Bristol, United Kingdom

**Keywords:** elevated CO_2_, stomatal movement, plant hormones, reactive oxygen species, NADPH oxidases, cell wall peroxidases

## Abstract

Elevated CO_2_ (eCO_2_) often reduces leaf stomatal aperture and density thus impacts plant physiology and productivity. We have previously demonstrated that the Arabidopsis BIG protein distinguishes between the processes of eCO_2_-induced stomatal closure and eCO_2_-inhibited stomatal opening. However, the mechanistic basis of this action is not fully understood. Here we show that eCO_2_-elicited reactive oxygen species (ROS) production in *big* mutants was compromised in stomatal closure induction but not in stomatal opening inhibition. Pharmacological and genetic studies show that ROS generated by both NADPH oxidases and cell wall peroxidases contribute to eCO_2_-induced stomatal closure, whereas inhibition of light-induced stomatal opening by eCO_2_ may rely on the ROS derived from NADPH oxidases but not from cell wall peroxidases. As with JA and ABA, SA is required for eCO_2_-induced ROS generation and stomatal closure. In contrast, none of these three signals has a significant role in eCO_2_-inhibited stomatal opening, unveiling the distinct roles of plant hormonal signaling pathways in the induction of stomatal closure and the inhibition of stomatal opening by eCO_2_. In conclusion, this study adds SA to a list of plant hormones that together with ROS from distinct sources distinguish two branches of eCO_2_-mediated stomatal movements.

## Introduction

Stomata formed by a pair of guard cells regulate gas exchanges between plants and the atmosphere. Guard cells sense and integrate both extra- and intracellular signals, such as light, temperature, carbon dioxide (CO_2_), plant hormones, leading to plant adaptive responses (Hetherington and Woodward, 2003; Murata et al., 2015; He and Liang, 2018). The continuing rise of atmospheric CO_2_ can profoundly impact plant physiology and crop yield potential via stomata, as elevated CO_2_ (eCO_2_) concentration in the atmosphere reduces leaf stomatal aperture and density in many species including crop plants (Woodward, 1987; Assmann, 1993; Keenan et al., 2013; Xu et al., 2016). Understanding CO_2_ signaling in guard cells is important in the context of breeding “climate change ready” crop varieties with improved agricultural performance and nutritional content (Kim et al., 2010; Myers et al., 2014; Caine et al., 2018; Zhang et al., 2018). In guard cells, CO_2_ signaling starts from CO_2_ conversion to bicarbonate (HCO_3_^–^) by βCA1 (beta Carbonic Anhydrase 1) and βCA4, followed by activation of MATE type transporter RHC1 (Resistance to High CO_2_), MPK4 (Mitogen-Activated Protein Kinase 4) and MPK12, subsequently leading to inhibition of HT1 (High Leaf Temperature 1), which phosphorylates and inactivates OST1 (Open Stomata 1). Repression of HT1 facilitates S-type anion channel activation by OST1 to mediate the anion effluxes resulting in stomatal closure (Hashimoto et al., 2006; Hu et al., 2010; Tian et al., 2015; Hashimoto-Sugimoto et al., 2016; Hõrak et al., 2016; Jakobson et al., 2016; Tõldsepp et al., 2018; Zhang et al., 2018).

As typified by the abscisic acid (ABA) receptors, the components in the stomatal closure induction and the stomatal opening inhibition are not necessarily the same (Assmann, 1993; Mishra et al., 2006; Yin et al., 2013; Dittrich et al., 2019). We have recently identified the Arabidopsis BIG protein as a novel component involved in eCO_2_-induced stomatal closure but not of eCO_2_-inhibited light-induced stomatal opening (He et al., 2018). BIG is involved in diverse processes including auxin transport, light and hormonal signaling, vesicle trafficking, endocytosis, phosphate deficiency tolerance, and the dynamic adjustment of circadian period (Li et al., 1994; Ruegger et al., 1997; Gil et al., 2001; Kanyuka et al., 2003; López-Bucio et al., 2005; Paciorek et al., 2005; Yamaguchi et al., 2007; Hearn et al., 2018). Mutations in the Arabidopsis *BIG* gene suppress eCO_2_-induced stomatal closure due to the disrupted activity of S-type ion channels (He et al., 2018). Direct channel regulation has been demonstrated to be insufficient to explain the strong eCO_2_-induced stomatal closing response in Arabidopsis (Wang et al., 2016). More recently, it has been shown that *big* mutants are more susceptible to bacterial pathogens that gain entry to the plant through stomata (Zhang et al., 2019). These findings point to the need to gain a better understanding of how BIG distinguishes two distinct processes of stomatal movement in response to eCO_2_. Given that reactive oxygen species (ROS) play a significant role in various signaling processes, and the results of investigations have revealed a role for BIG in redox signaling (Rhee et al., 2000; Gil et al., 2001; Grek et al., 2013; Song et al., 2014; Parsons et al., 2015; Zhang et al., 2019), we hypothesized that ROS production has a central role to play in defining stomatal responses to eCO_2_.

ROS including hydrogen peroxide (H_2_O_2_) and superoxide (O_2_^–^) are widely produced in different cellular compartments in plants and have been recognized as a major regulator in various aspects of plant life such as stomatal development and movement, particularly under different abiotic and biotic stress conditions (McAinsh et al., 1996; Neill et al., 2002; Foyer and Noctor, 2005; Song et al., 2014; Sierla et al., 2016). In Arabidopsis, apoplastic ROS are mainly produced by plasma membrane-localized NADPH oxidases and cell wall peroxidases (Song et al., 2014; Murata et al., 2015; Singh et al., 2017), and the activities of these different types of enzymes are strongly inhibited by diphenylene iodonium (DPI) and salicylhydroxamic acid (SHAM), respectively (Allan and Fluhr, 1997; Pei et al., 2000; Mori et al., 2001; Khokon et al., 2011; Miura et al., 2013). The evolution and maintenance of different sources for ROS production is most likely due to the requirement for intricate control of oxidative signaling, given the fact that ROS can be cytotoxic and mutagenic and for their proper function in signaling their production must be tightly regulated both temporally and spatially (Mittler, 2017).

ABA and jasmonate (JA) induce ROS accumulation in guard cells via the activities of two NADPH oxidases, RBOHD and RBOHF (Torres et al., 2002, 2006; Kwak et al., 2003; Suhita et al., 2004), whereas salicylic acid (SA) likely regulates ROS homeostasis via the peroxidases-catalyzed reactions (Mori et al., 2001; Khokon et al., 2011), and the inhibition of catalase and ascorbate peroxidase (Chen et al., 1993; Durner and Klessig, 1995). eCO_2_-induced stomatal closure is suppressed in the *rbohDrbohF* double mutants (Kolla et al., 2007; Chater et al., 2015). Peroxidases are bifunctional enzymes, through two possible catalytic cycles, hydroxylic and peroxidative, to generate or detoxify and regulate H_2_O_2_ levels. For example, during the hydroxylic cycle, the peroxidases catalyze the generation of ⋅OH and HOO⋅ from H_2_O_2_ by two different routes (Passardi et al., 2004). In Arabidopsis, there are 73 isoforms of cell wall peroxidases (Tognolli et al., 2002; Passardi et al., 2006). Two cell wall peroxidase-encoding genes, *PRX33* and *PRX34*, which are highly and preferentially expressed in guard cells compared with other PRXs members according to Genevestigator (an available microarray database^1^), are widely involved in H_2_O_2_ production against fungi-, bacteria-, SA-, and flg22-induced stomatal closure (Bindschedler et al., 2006; Daudi et al., 2012; O’Brien et al., 2012a,b; Arnaud et al., 2017). Notably, SA-mediated ROS production and stomatal closure are not impaired by DPI or in *rbohDrbohF* double mutant (Khokon et al., 2011). In contrast to NADPH oxidases, the importance of ROS-producing peroxidases to plant adaptive responses, particularly their function in regulating eCO_2_-mediated stomatal movement, has largely been overlooked.

In this study, by combining pharmacological and genetic approaches, we reveal distinct roles of ROS-producing peroxidases and NADPH oxidases for eCO_2_-induced stomatal movements. We also found that endogenous SA and SA-signaling components are required for eCO_2_-induced stomatal closure. Neither ABA, JA, or SA are involved in regulating eCO_2_-inhibited stomatal opening. In conclusion, our data suggest that plant hormones and ROS from distinct sources selectively mediate different stomatal CO_2_ responses, and shed new light on ROS action and the CO_2_ signaling network.

## Materials and Methods

### Plant Material and Growth Conditions

All Arabidopsis (*Arabidopsis thaliana* L.) lines used in this study were in the Columbia background (Col-0). Seeds of *sid2-2*, *npr1-1*, *npr3npr4*, and *rbohDrbohF* were kindly provided by Drs Shunping Yan and Honghong Hu (Huazhong Agricultural University, China). Seeds of *prx33-3* and *prx34-2* was a gift from Dr. Ildoo Hwang (Pohang University of Science and Technology, Korea). More information of the mutants used in this study are shown in Supplementary Table S1. Seed germination and plant growth were essentially carried out as described in He et al. (2018). For stomatal aperture bioassays, seeds were surface-sterilized and sown on half-strength Murashige and Skoog (MS) medium plates containing 0.6% agar and 1% sucrose. After stratification (4°C in the dark for 2 days), the plates were transferred to the green house at 22°C/18°C (day/night) with 10 h/14 h (light/dark) photoperiod cycle (light intensity 120 μ moles photons m^–^^2^s^–^^1^), 50% relative humidity, at ambient CO_2_, approximate 450 ppm. Ten days old plants were transferred to soil and grown in the same green house for the future experiments. For the stomatal bioassays, 4–5 weeks old plants were used.

### Stomatal Aperture Measurements

For elevated CO_2_-induced stomatal closure, abaxial epidermis of fully expanded leaves were detached and incubated for 2.5 h under 150 μmol m^–^^2^s^–^^1^ light in 50 mM KCl, 10 mM MES/KOH (pH 6.15) at 22°C whilst being aerated with CO_2_-free air by bubbling through the buffer solution to bring about stomatal opening, and then either aerated with CO_2_-free air or elevated CO_2_ (800 ppm) for additional 2.5 h before peels were removed, mounted on slides and stomatal aperture measurements were recorded using an inverted microscope (Olympus BX51), fitted camera (Olympus DP70), and ImageJ software v. 1.43u (NIH). For inhibition of stomatal opening, epidermal peels of abaxial epidermis floated on the 10 mM MES/KOH (pH 6.20) in 6 cm dishes at 22°C for 1 h under the dark, then directly transferred to fresh dishes and incubated for 3 h under light of 150 μmol m^–^^2^s^–^^1^ in 50 mM KCl, 10 mM MES/KOH (pH 6.15) at 22°C either aerated with CO_2_-free air or elevated CO_2_ (800 ppm) by bubbling directly into the buffer.

Details of the DPI, SHAM and Tiron treatments were as follows: The ROS scavenger Tiron (4,5-dihydroxy-1,3-benzenedisulfonic acid) (Sigma-Aldrich, United States) was dissolved in water and used at a final concentration of 10 mM, ROS inhibitor DPI (diphenyl iodonium chloride) (Sigma-Aldrich, United States) was dissolved in DMSO and used at a final concentration of 20 μM, SHAM (salicylhydroxamic acid) (Sigma-Aldrich, United States) was dissolved in ethanol and used at a final concentration of 2 mM, these chemicals were added 30 min prior to the addition of 800 ppm CO_2_. The highest concentration of DMSO or ethanol that was used was added to the zero treatments as a control. To avoid experimenter bias, all the aperture measurements were performed blind. Forty or sixty stomatal apertures were measured per treatment and measurements from two replicates of each treatment were pooled and analyzed by GraphPad Prism 8.0.2 (GraphPad).

### Measurement of ROS Production in Guard Cells

2′,7′-Dichlorofluorescein diacetate (H_2_DCF-DA) (Sigma-Aldrich, United Kingdom) fluorescence was used as a measure for ROS levels as previously described (Chater et al., 2015). Briefly, epidermal peels from treated leaves were incubated in 50 mM KCl, 10 mM MES/KOH (pH 6.15) buffer in the presence of 50 μM H_2_DCF-DA for 10 min at 22°C in darkness. Epidermal strips were washed with 50 mM KCl, 10 mM MES/KOH (pH 6.15) buffer at room temperature. Subsequently, the fluorescence in guard cells was detected using TCS-SP8 confocal laser scanning microscope (Leica lasertechnik GmbH, Heidelberg, Germany). The fluorescent intensities of each image were analyzed using Photoshop 7.0 (ASI). At least fifty guard cell pairs were measured per experiment and analyzed by GraphPad Prism 8.0.2 (GraphPad). To avoid experimenter bias, all the fluorescent intensities measurements were performed blind. Each experiment was done at least three independent times with similar results.

### Gene Expression Analysis by Quantitative Real-Time PCR

Total RNA from aerial parts of the plants was extracted using RNeasy^®^ total RNA mini kit (Qiagen) followed by plant genomic DNA digestion with RNase-free DNase I (Thermo scientific) according to the manufacturer’s instructions. The absence of genomic DNA contamination was confirmed by PCR using RNA as template without reverse transcription. First strand cDNA was synthesized using Superscript II^®^ reverse transcriptase (Invitrogen) and oligo d(T)_15__–__18_ (Promega) mRNA primer with 1 μg of total RNA as the template. cDNA corresponding to 20 ng of total RNA and 300 nM of each primer were used in PCR reactions. Primer sequences used for RT-PCR and quantitative RT-PCR are listed in Supplementary Table S2. Experiments on independently grown plant material were carried out three times and data analyzed by GraphPad Prism 8.0.2 (GraphPad).

### Statistical Analysis

The data were statistically analyzed using GraphPad Prism 8.0.2 (GraphPad). The effects of CO_2_ and chemical treatments as well as their interactions on variables were analyzed using analysis of variance (ANOVA). Differences between treatments were considered significant when the *P*-value was less than 0.05 by Tukey’s test.

## Results

### Cell Wall Peroxidases and NADPH Oxidases Are Required for eCO_2_-Induced Stomatal Closure

To test the hypothesis that ROS production has a central role to play in defining stomatal CO_2_ responses, we started by monitoring ROS levels in the *big* mutant and wild-type Col-0 (WT) plants using the fluorescence of H_2_-DCFDA. As shown in Supplementary Figure S1A, the application of eCO_2_ (800 ppm) resulted in rapid enhancement of fluorescence in WT guard cells, whereas the increases of ROS were greatly reduced in all *big* mutant alleles examined, including *big-1*, *doc1-1*, and *big-j588* (Supplementary Figure S1A), consistent with the compromised eCO_2_-induced stomatal closure (He et al., 2018). Strikingly, during eCO_2_ inhibited light-induced stomatal opening, we observed comparable increases of ROS levels in the guard cells of the *big* mutant and WT plants (Supplementary Figure S1B), in line with previous results (He et al., 2018). These data suggest that CO_2_-stimulated stomatal closure and inhibition of light-induced opening both employ an increase in ROS. This suggests that the guard cells might employ different mechanisms to discriminate the types and strength of ROS signals and thereby finely tune stomatal movements in response to eCO_2_.

**FIGURE 1:**
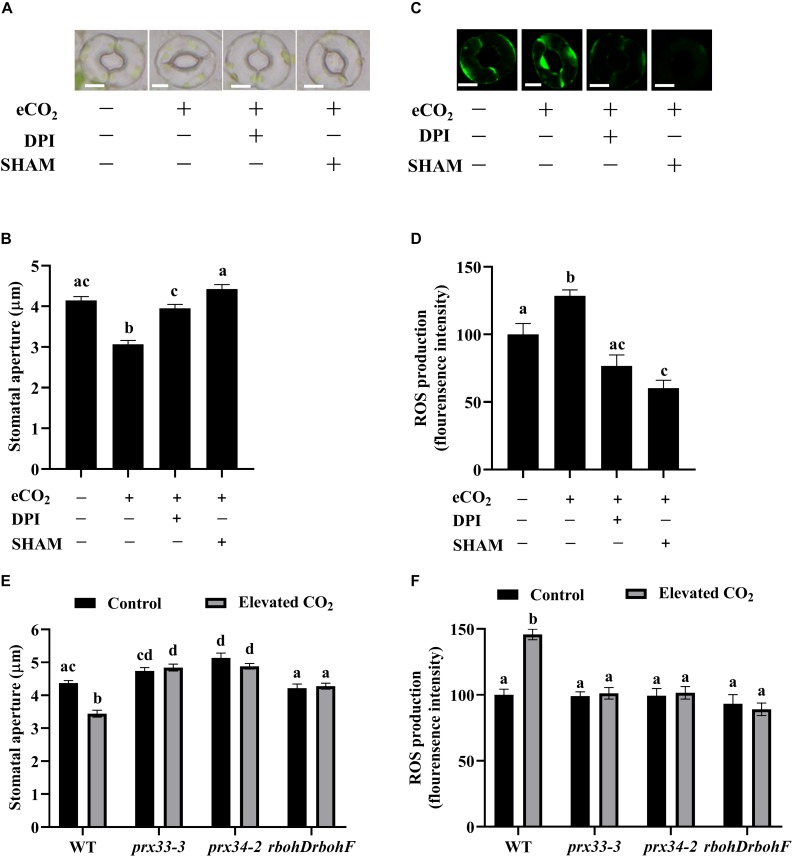
Cell wall peroxidases and NADPH oxidases are required for elevated CO_2_-induced stomatal closure. **(A)** eCO_2_-induced stomatal closure is inhibited by ROS inhibitors DPI and SHAM. Representative images showing guard cells of WT: after 2.5 h light-incubation, epidermal peels of WT plants were treated with 800 ppm CO_2_ for another 2.5 h before photos taken. 20 μM DPI or 2 mM SHAM added before CO_2_ treatment for 30 min. Scale bar, 5 μm. **(B)** Quantitative stomatal aperture from (**A**). **(C)** Representative images showing H_2_DCF-DA fluorescence of WT guard cells under control (CO_2_-free air) and elevated (800 ppm) CO_2_ with or without ROS inhibitors DPI or SHAM treatment. Scale bar, 5 μm. **(D)** Quantitative ROS production from **(C)**. eCO_2_ stimulates an increase of H_2_DCF-DA fluorescence in guard cells that is blocked in the presence of DPI/SHAM. **(E)** eCO_2_-induced stomatal closure is disrupted in *prx33-3*, *prx34-2*, and *rbohDrbohF* mutants. **(F)** eCO_2_-induced ROS production in guard cells is compromised in *prx33-3*, *prx34-2*, and *rbohDrbohF* mutants during stomatal closure. In **(B)** (*n* = 120), **(D)** (*n* = 50), **(E)** (*n* = 80), and **(F)** (*n* = 60), values are means ± s.e. All experiments were repeated at least three times. Different letters represent statistically significant differences at *P* < 0.05 based on a Tukey’s test.

The functioning of NADPH oxidases RBOHD and RBOHF in eCO_2_-induced stomatal closure has been well documented (Kolla et al., 2007; Chater et al., 2015; Geng et al., 2016), while the function of cell wall peroxidases in CO_2_ signaling remains to be investigated. Figures 1A,B show that eCO_2_ caused an average 25% reduction in stomatal apertures, whereas this reduction was efficiently abolished by either NADPH oxidases inhibitor DPI or cell wall peroxidases inhibitor SHAM. Around 30% extra ROS were induced by eCO_2_ treatments, but peels pre-treated with DPI or SHAM failed to exhibit significant ROS accumulation during eCO_2_ treatment (Figures 1C,D). These results suggest that cell wall peroxidases function in eCO_2_-induced stomatal closure. We next examined the stomatal CO_2_ responses in *prx33-3* and *prx34-2* using the *rbohDrbohF* double mutants as a positive control. Similar to *rbohDrbohF*, stomatal apertures of both *prx33-3* and *prx34-2* mutant lines failed to close in response to eCO_2_ (Figure 1E). In line with this observation, ROS accumulation was not triggered by eCO_2_ in the *prx33-3*, *prx34-2*, or *rbohDrbohF* mutants in marked contrast to an over 50% ROS increase in WT (Figure 1F). These data not only support the notion that CO_2_-induced stomatal closure is dependent on ROS (H_2_O_2_) production (Kolla et al., 2007; Chater et al., 2015; Geng et al., 2016), but also demonstrate an essential role of cell wall peroxidases including PRX33 and PRX34 in response to eCO_2_, shedding new light on ROS action in plants. Furthermore, as with two eCO_2_ inducible genes, *SLAC1* and *OST1* (Shi et al., 2015; Dittrich et al., 2019), expressions of *RBOHD*, *RBOHF*, *PRX33*, and *PRX34* were upregulated by eCO_2_ (Supplementary Figure S2), further corroborating our view that both NADPH oxidases and cell wall peroxidases function in guard cell eCO_2_ signaling.

### eCO_2_-Mediated Stomatal Opening Inhibition Requires ROS Generation

**FIGURE 2:**
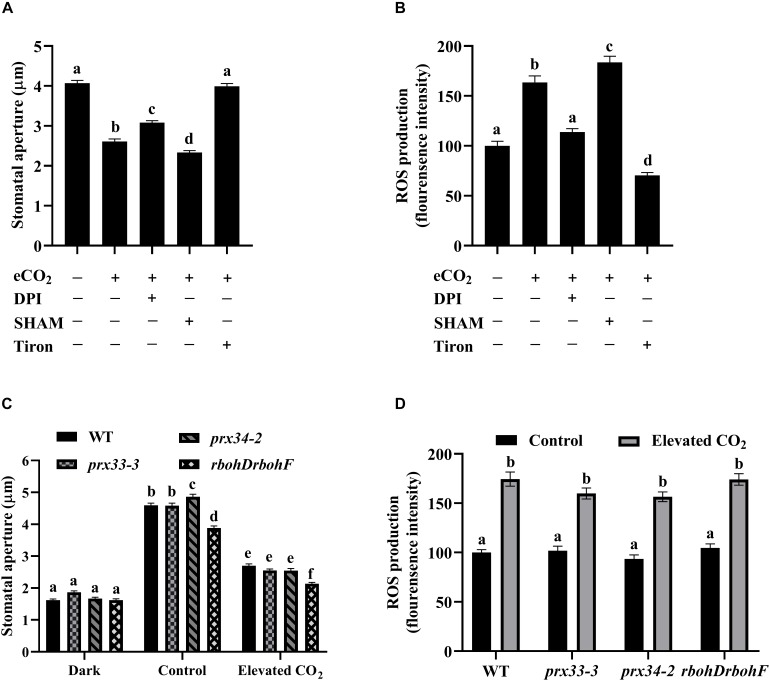
The inhibition of light-induced stomatal opening by eCO_2_ requires ROS generation. **(A)** eCO_2_-inhibited stomatal opening is compromised by treatment with Tiron. Stomatal apertures were measured on light-incubated epidermal peels treated with CO_2_-free (mock) or 800 ppm CO_2_ (elevated CO_2_) for 3 h. DPI, SHAM, and Tiron added before light treatment for 30 min. **(B)** eCO_2_ stimulates an increase of H_2_DCF-DA fluorescence in guard cells that is blocked in the presence of DPI and Tiron. **(C)** eCO_2_-inhibited stomatal opening in *prx33-3*, *prx34-2*, and *rbohDrbohF* mutants is similar to WT. Stomatal apertures were measured on illuminated epidermal peels treated with CO_2_-free (mock) or 800 ppm CO_2_ (elevated CO_2_) for 3 h. Dark represents 1 h dark-adapted stomata incubated in the 10 mM MES/KOH (pH 6.20) buffer. **(D)** eCO_2_ stimulates an increase in guard cells of H_2_DCF-DA fluorescence in WT as well as in *prx33-3*, *prx34-2*, and *rbohDrbohF* mutants. Mean fluorescence intensity was measured on light-incubated epidermal peels treated with CO_2_-free (mock) or 800 ppm CO_2_ (elevated CO_2_) for 3 h. In **(A)** (*n* = 120), **(B)** (*n* = 60), **(C)** (*n* = 120), and **(D)** (*n* = 60), values are mean ± s.e. All experiments were repeated at least three times. Different letters represent statistically significant differences at *P* < 0.05 based on a Tukey’s test.

eCO_2_-induced stomatal closure and the inhibition of light-induced stomatal opening by eCO_2_ are two separate processes (He et al., 2018). Figure 1 shows that eCO_2_-induced stomatal closure requires ROS from both NADPH oxidases and cell wall peroxidases. In eCO_2_-inhibited light-induced stomatal opening, eCO_2_ suppressed opening induced by 36% and this was associated with an approximate 60% greater increase in ROS accumulation compared to mock treated plants (Figures 2A,B). eCO_2_-inhibited light induced stomatal opening was virtually abolished by Tiron, a potent ROS scavenger (Figure 2A; Yamada et al., 2003). Consistently, the eCO_2_-induced ROS accumulation was inhibited by Tiron (Figure 2B). Together, these data support the hypothesis that ROS production is indispensable to eCO_2_-mediated inhibition of stomatal opening. DPI dampened stomatal opening inhibition presumably by blocking eCO_2_-induced ROS increase, as in the presence of DPI, a 24% reduction in stomatal aperture accompanied with a slight while statistically insignificant increase (14%) of ROS production was observed (Figures 2A,B). However, neither eCO_2_-inhibited stomatal opening nor eCO_2_-induced ROS accumulation was compromised by SHAM (Figures 2A,B). The inhibition of stomatal opening by eCO_2_ required ROS accumulation which might be dependent on NADPH oxidases but less likely on cell wall peroxidases. These data suggest that ROS from distinct sources differentially modulate eCO_2_-triggered stomatal movements. Importantly, when the *rbohDrbohF*, *prx33-3*, and *prx34-2* and WT plants were analyzed, we observed similar eCO_2_*-*inhibited stomatal opening and guard cell ROS accumulations (Figures 2C,D), suggesting RBOHD, RBOHF, PRX33, and PRX34 are unlikely to be involved in the inhibition of stomatal opening by eCO_2_. On the basis of our results we conclude that sources of ROS, other than those described above, must be involved in eCO_2_-inhibited stomatal opening.

### eCO_2_-Induced ROS and Stomatal Closure Require SA and SA Signaling

**FIGURE 3:**
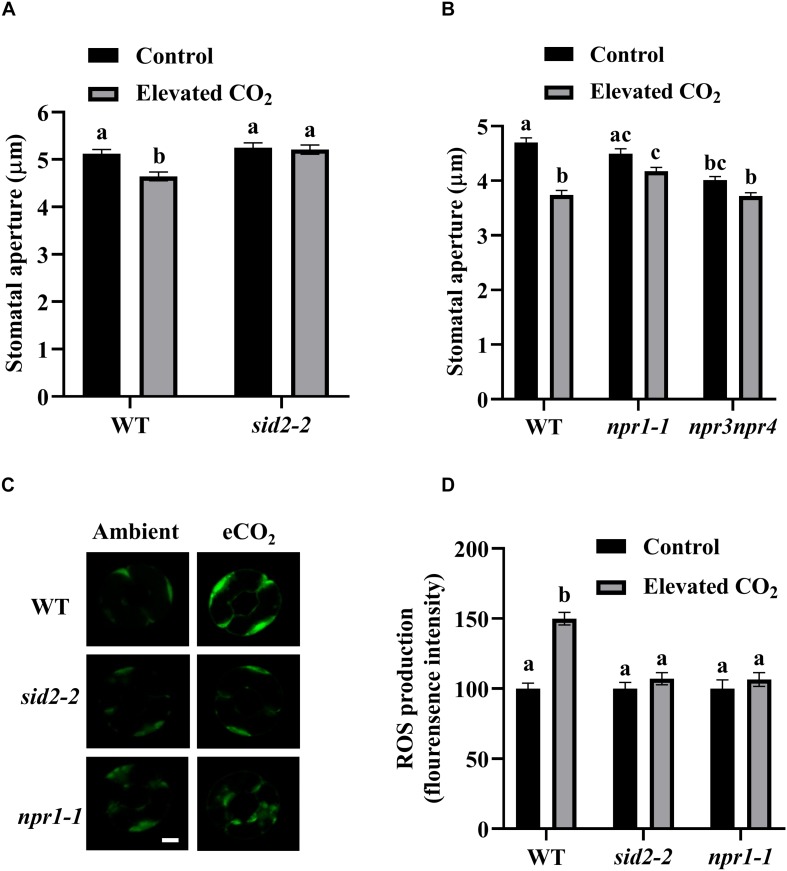
eCO_2_-induced stomatal closure requires SA and SA signaling. **(A)** eCO_2_-induced stomatal closure is corrupted in *sid2-2* mutants. **(B)** eCO_2_-induced stomatal closure is disrupted in *npr1-1* and *npr3npr4* mutants. **(C)** Representative images showing H_2_DCF-DA fluorescence of WT, *sid2-2* and *npr1-1* mutants guard cells under control (CO_2_-free air) and elevated (800 ppm) CO_2_. Scale bar, 5 μm. **(D)** eCO_2_ stimulates an increase of H_2_DCF-DA fluorescence in WT guard cells, but is blocked in *sid2-2* and *npr1-1* mutants. Mean fluorescence intensity was measured on 2.5 h light-preincubated epidermal peels, treated with 800 ppm CO_2_ for another 2.5 h. Stomatal apertures in **(A,B)** were measured on 2.5 h light-preincubated epidermal peels, treated with 800 ppm CO_2_ for another 2.5 h. In **(A)** (*n* = 120), **(B)** (*n* = 120), and **(D)** (*n* = 60), values are mean ± s.e. All experiments were repeated at least three times. Different letters represent statistically significant differences at *P* < 0.05 based on a Tukey’s test.

SA can modulate plant growth, development and responses to a wide range of biotic and abiotic stresses. To determine whether SA participates in eCO_2_-induced stomatal closure, we measured stomatal apertures and ROS production using SA-deficient mutant *sid2-2* (SA Induction-Deficient 2) after eCO_2_ treatments. While stomatal apertures of WT were reduced by about 10%, no significant reduction of stomatal apertures was detected in *sid2-2* by eCO_2_ application (Figure 3A). Additionally, we tested *npr1-1*, *npr3npr4* mutants because NPR1, NPR3, and NPR4 are key components of SA signaling (Fu et al., 2012; Wu et al., 2012; Kuai et al., 2015; Ding et al., 2018). In contrast to a nearly 20% reduction of stomatal apertures in WT, *npr1-1* and *npr3npr4* mutants displayed no appreciable eCO_2_-induced stomatal closure (Figure 3B). Consistently, eCO_2_-induced ROS accumulation in guard cells was completely abolished in *sid2-2*, *npr1-1* as well as in *npr3npr4* (Figures 3C,D and Supplementary Figure S3). These results indicate that eCO_2_-induced stomatal closure requires an intact SA signaling pathway, and both SA biosynthesis and SA signaling are involved in eCO_2_-induced ROS production.

### SA, JA, and ABA Function Differently in eCO_2_-Inhibited Stomatal Opening

**FIGURE 4:**
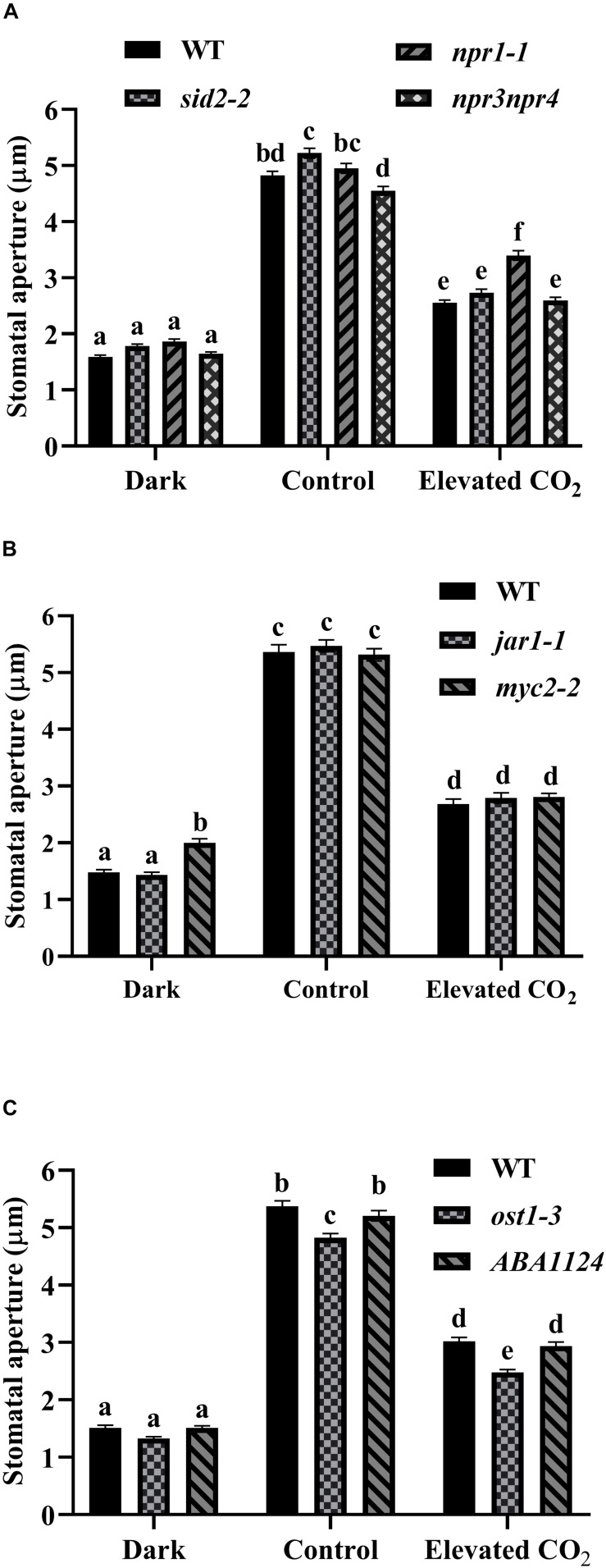
SA, JA, and ABA are not required for the inhibition of light-stimulated stomatal opening by eCO_2_. **(A)** eCO_2_-inhibited stomatal opening in *sid2-2*, *npr1-1*, and *npr3npr4* mutants is similar to WT. **(B)** eCO_2_-inhibited stomatal opening in *jar1-1* and *myc2-2* mutants is similar to WT. **(C)** eCO_2_-inhibited stomatal opening in *pyr1pyl1pyl2pyl4* (*ABA1124*) and *ost1-3* mutants is similar to WT. Stomatal apertures were measured on light-incubated epidermal peels treated with CO_2_-free (mock) or 800 ppm CO_2_ (elevated CO_2_) for 3 h. Dark represents 1 h dark-adapted stomata incubated in the 10 mM MES/KOH (pH 6.20) buffer. In **(A–C)** (*n* = 120), the shown result is a representative of three independent biological experiments, values are mean ± s.e. Different letters represent statistically significant differences at *P* < 0.05 based on a Tukey’s test.

As shown in Figure 4A, stomata of WT and *sid2-2*, *npr1-1*, and *npr3npr4* exhibited a similar degree of closure as WT after 1 h dark treatment. Light-induced stomatal opening in *sid2-2* and *npr3npr4* was similar to WT while apertures of *npr1-1* were consistently larger than WT (Figure 4A). When treated with eCO_2_, the reduction in stomatal aperture of either *sid2-2* (48%) or *npr3npr4* (43%) was similar to that of WT (47%), indicating that eCO_2_-inhibited stomatal opening was not compromised in *sid2-2* and *npr3npr4*, but partially impaired in *npr1-1* (31% reduction) (Figure 4A). Based on these results we conclude that SA biosynthesis and SA signaling play no significant role in eCO_2_-inhibited light-induced stomatal opening.

We next examined the involvement of ABA and JA signaling which has been reported to be essential for eCO_2_-induced stomatal closure (Chater et al., 2015; Geng et al., 2016; Hsu et al., 2018) in eCO_2_-inhibited stomatal opening. First we verified that JA pathway deficient mutants *coi1-1* and *jar1-1* are insensitive to eCO_2_-induced stomatal closure (Supplementary Figure S4). These data confirmed the results of Geng et al. (2016). *myc2-2*, a loss-of-function mutant line of *MYC2*, which is a master regulator of JA signaling, and *jar1-1*, behaved similarly to WT (47, 49, 50% reduction of stomatal aperture, respectively) in eCO_2_-inhibited light-induced stomatal opening (Figure 4B). Likewise, both the quadruple ABA receptor mutant *pyr1pyl1pyl2pyl4* (*ABA1124*) and *ost1-3* exhibited wild type (44, 49, 44% reduction of stomatal aperture, respectively) responses to eCO_2_-inhibited stomatal opening (Figure 4C). Taken together, it appears that ABA and JA signaling pathway are not directly involved in eCO_2_-inhibited light-induced stomatal opening.

## Discussion

### Different Sources of ROS Play Different Roles in eCO_2_-Induced Stomatal Movement

An increase in guard cell ROS, including H_2_O_2_ in response to diverse stimuli is one of the first measurable events in stomatal movements. H_2_O_2_ production mainly depends on two types of enzymes in guard cells, one is NADPH oxidases and the other is cell wall peroxidases (Murata et al., 2015). Similar to the bacterial pathogen-associated molecular patterns (PAMPs), flagellin (flg22) that induces stomatal closure, eCO_2_-induced stomatal closure requires both NADPH oxidases- and cell wall peroxidases-generated ROS (Figure 1; O’Brien et al., 2012b). Intriguingly, when we analyzed *RBOHD*, *RBOHF*, *PRX33*, and *PRX34* expression levels in *rbohDrbohF*, *prx33*, and *prx34* mutant plants, we found that loss of function of any individual gene had no detectable effects on the expression of the other genes (Supplementary Figure S5). In addition, the disruption of one gene is not compensated by other functional ROS generation related genes, indicating there is no feedback and/or counterbalancing regulations among the expressions of NADPH oxidases and cell wall peroxidases. This is consistent with the observation that the cytokinin analog trans-zeatin induces stomatal closure and ROS accumulation in guard cells involving the apoplastic PRXs PRX4, PRX33, PRX34, and PRX71, but not the NADPH oxidases RbohD and RbohF (Arnaud et al., 2017). Thus, it is highly possible that NADPH oxidases and cell wall peroxidases function independently to generate ROS during eCO_2_-/PAMP-induced stomatal closure. More dedicated experiments including the evaluation of the possible additive effects on ROS production between *prx33*/*34* and *rbohD*/*F* mutants will be needed to further assess this interpretation. Notably, it has been quantitatively determined that peroxidases are responsible for half of the ROS produced in response to PAMPs, while the other half is produced by NADPH oxidases and/or mitochondrial and chloroplastic ROS sources (O’Brien et al., 2012b).

In contrast to eCO_2_-induced stomatal closure, eCO_2_-inhibited stomatal opening was only partially blocked by DPI treatment but not by SHAM. These results are in line with the insights we got from working with BIG (Supplementary Figure S1), namely, that guard cells employ different mechanisms to discriminate the types and strength of ROS signals in order to, presumably, finely tune stomatal movements in response to eCO_2_. Interestingly, a recent paper reported that neither DPI nor SHAM reduced the high level of ROS in the *atg*2 mutant, which is compromised in light- and low CO_2_-induced stomatal opening (Yamauchi et al., 2019). While both eCO_2_-inhibited stomatal opening and ROS accumulation could be entirely abrogated by Tiron, the inhibition of stomatal opening by eCO_2_ remains functional in the *rbohDrbohF* double mutant (Figure 2). This suggests that other ROS sources, which are inhibited by Tiron but not by DPI function in eCO_2_-inhibited light-induced stomatal opening. Nitric oxide (NO) which plays a role in stomatal movement (Neill et al., 2003; Laxalt et al., 2016) has been identified to be required for eCO_2_-induced stomatal closure in tomato (Shi et al., 2015). Evidently, NO production might also contribute to eCO_2_-triggered stomatal movement in Arabidopsis. In addition to NADPH oxidases and cell wall peroxidases, the polyamine oxidases (PAOs) catalyze catabolism of spermidine and spermine with concomitant production of H_2_O_2_ (Pottosin et al., 2014; Sierla et al., 2016). An inhibitor of PAOs interferes with ABA-induced stomatal closure in French bean and ethylene-induced stomatal closure in Arabidopsis (An et al., 2008; Hou et al., 2013). Whether and how PAOs contribute to the ROS accumulations that drive eCO_2_-reguated stomatal movement remains to be investigated. Another possibility is that other members of the NADPH oxidase family function in guard cell signaling in response to eCO_2_. In Arabidopsis, there are 10 members of the RBOH family. When the spatiotemporal expression profile of all *RBOH* members is examined using ePlant^2^, it is apparent that, in addition to *RBOHD* and *RBOHF*, *RBOHC* is highly expressed in guard cells (Supplementary Figure S6), suggesting a regulatory role of RBOHC in stomatal function. This suggestion is supported by work from Wei et al. (2018) who provided evidence that the activity of RBOHC is required for melatonin-induced stomatal closure and ROS production. It will be interesting to investigate whether RBOHC is involved in eCO_2_-induced stomatal movement.

Apoplastic ROS are known to regulate stomatal movement, however they are sensed and transduced is not well understood. One possibility is that apoplastic ROS are sensed by yet to be characterized extracellular sensors and subsequently transduced by unknown intracellular pathways (Sierla et al., 2016). Alternatively, apoplastic ROS such as H_2_O_2_ can be transported into the cytoplasm via aquaporins (Tian et al., 2016), as exemplified by the aquaporin PIP2;1 which is required for ABA and flg22-induced H_2_O_2_ accumulation in guard cells (Rodrigues et al., 2017). Moreover, ROS can directly modify the activity of ion channels leading to stomatal closure (Pei et al., 2000). Equally possible, however, is that ROS function through parallel mechanisms to promote CO_2_ signaling in guard cells.

### Plant Hormone Signals Differentially Mediate eCO_2_-Regulated Stomatal Movement

Changes in SA concentration after pathogen infection affect the redox state of the cell and bring about a conformational switch of NPR1 (Cao et al., 1994) and thereby activate *PR* genes (Chen et al., 1993; Vanacker et al., 2000; Noctor et al., 2002; Mou et al., 2003). eCO_2_ can induce SA production and activate SA signaling in many plant species (Matros et al., 2006; Casteel et al., 2012; Huang et al., 2012; Zhang et al., 2015; Mhamdi and Noctor, 2016; Williams et al., 2018). Our observation that eCO_2_-induced stomatal closure requires endogenous SA and SA-signaling components supports a proposed link between SA and CO_2_ signaling in guard cells response (Medina-Puche et al., 2017). This is in line with several reports that SABP3 (SA-binding protein 3), a chloroplast carbonic anhydrase (CA), which exhibits both CA enzymatic and SA-binding activities is indispensable to SA-mediated defense response in tomato as well as in Arabidopsis (Slaymaker et al., 2002; Wang et al., 2009). Also, NPR1 and NRB4 (Non-recognition of BTH 4, another SA signaling component) interact with βCA1 (Medina-Puche et al., 2017). In addition, it is known that the β*ca1*β*ca4* double mutant compromises CO_2_ sensing (Hu et al., 2010). These, together with the fact that the quintuple mutant β*ca1*β*ca2*β*ca3*β*ca4*β*ca6* shows reduced sensitivity to SA, suggest that CAs likely function in modulating the perception of SA in plants (Medina-Puche et al., 2017). Although NPR1 and NPR3/NPR4 play opposite roles in transcriptional regulation, they all function in a SA-dependent manner for plant immune responses as NPR1, NPR3, and NPR4 are SA-binding proteins (Ding et al., 2018). Nevertheless, both *npr1-1* and *npr3npr4* are insensitive to eCO_2_-induced stomatal closure (Figure 3), in line with the finding that the double mutant *npr3npr4* is defective in systemic acquired resistance (SAR) (Fu et al., 2012), suggesting disruption in different aspects of SA signaling components might consequently affect eCO_2_-induced stomatal closure. Interestingly, eCO_2_-inhibited stomatal opening was partially compromised only in *npr1-1* but not in *sid2-2* or *npr3npr4* (Figure 4A). It is assumed that selective SA-binding to NPR1 and NPR3/NPR4 could differentially affect eCO_2_-inhibited stomatal opening. Alternatively, SA-independent NPR1 function in ER (endoplasmic reticulum) stress has been reported recently (Lai et al., 2018), thus NPR1 might function in a SA-independent manner during eCO_2_-inhibited stomatal opening. PRX33 and PRX34 play a significant role in SA-mediated stomatal closure (Arnaud et al., 2017). Our observation that SA signaling pathway functions in eCO_2_-induced stomatal closure rather than in eCO_2_-inhibited stomatal opening (Figures 3, 4), is in accordance with that cell wall peroxidases differentially mediate eCO_2_-regulated stomatal movement (Figures 1, 2), implicating that SA regulates eCO_2_ inhibition of stomatal closure via the activities of the peroxidases, which needs to be assessed in more details in the future, for example, by examining the expression changes of PRX33 and PRX34 in response to eCO_2_ in the SA mutants using RBOHD and RBOHF as experimental controls.

Multiple lines of evidence support a requirement of ABA for perceiving CO_2_ concentration changes by stomata (Raschke, 1975; Webb and Hetherington, 1997; Merilo et al., 2013; Chater et al., 2015; Hsu et al., 2018). Recently, Dittrich et al. (2019) have further demonstrated that ABA receptors PYL4 and PYL5 are key to CO_2_-induced stomatal closure. JA and SA signaling pathways are often mutually antagonistic, which can be induced simultaneously under eCO_2_ and intracellular oxidative stresses (Han et al., 2013a, b; Mhamdi and Noctor, 2016; Williams et al., 2018). The present study shows that both SA and JA are required for mediating stomatal closure by eCO_2_ (Figure 3 and Supplementary Figures S5), in line with the emerging evidences that SA, JA, ABA and ROS signaling are important in linking CO_2_ availability, stomatal function and the activation of plant defense responses (Li et al., 2014; Geng et al., 2016; Mhamdi and Noctor, 2016; Zhou et al., 2017; Williams et al., 2018). To further substantiate these findings, the contents of SA, JA and ABA need to be monitored in the future experiments. However, there is no evidence that eCO_2_ brings about an elevation of ABA (Chater et al., 2015; Hsu et al., 2018). ABA and JA can induce ROS accumulation in guard cells via the activities of RBOHD and RBOHF, whereas SA regulates ROS homeostasis via the peroxidases-catalyzed reactions (Murata et al., 2015). ABA, JA and SA are known to be required for eCO_2_-induced stomatal closure. However, our data indicate that none of these three hormones plays major roles in eCO_2_-inhibited stomatal opening, a process that is dependent on ROS generation, reflecting a similar mechanism in O_3_-induced ROS stress responses which are independent on SA, JA and ethylene signals (Xu et al., 2015).

**FIGURE 5:**
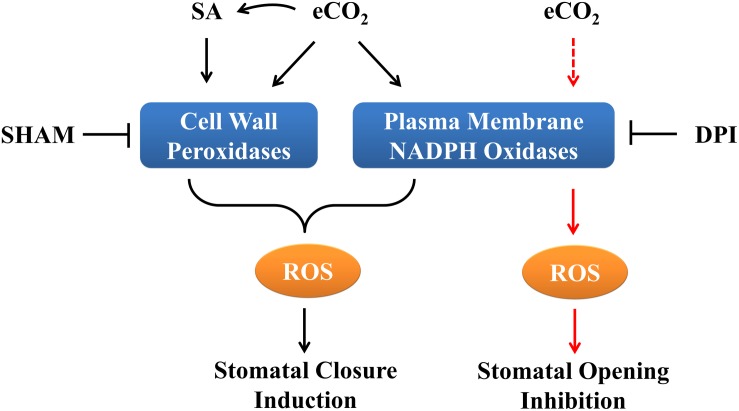
Schematic diagram of ROS accumulation and stomatal movement in response to eCO_2_ in Arabidopsis. In terms of cell wall peroxidases and NADPH oxidases it appears both of which function in eCO_2_-induced stomatal closure. SA signaling is required for eCO_2_-regulated stomatal closure, while eCO_2_-inhibited stomatal opening may only depend on NADPH oxidases mediated ROS generation. Black lines signify induced stomatal closure and red lines signify inhibited stomatal opening. Solid lines indicate verified interactions; dashed lines indicate hypothetical interactions.

In this study, by investigating ROS accumulation and stomatal movement in response to eCO_2_, we demonstrated that both cell wall peroxidases and NADPH oxidases are required for ROS production during eCO_2_-mediated stomatal closure, whereas eCO_2_-inhibited stomatal opening might be dependent on NADPH oxidases but not on cell wall peroxidases (Figure 5). The data presented here indicate that eCO_2_-inhibited light-stimulated stomatal opening requires ROS. However, our data suggest that distinct sources of ROS other than NADPH oxidases and PRXs play vital roles in stomatal opening inhibition by eCO_2_. Furthermore, we show that as with JA and ABA, SA signals are required for eCO_2_-induced stomatal closure and ROS generation. None of these three hormones has a significant role in eCO_2_-inhibited stomatal opening. Taken together, these results suggest that ROS from distinct sources and various plant hormones differentially regulate eCO_2_-induced stomatal movement.

## Data Availability Statement

All datasets generated for this study are included in the article/Supplementary Material.

## Author Contributions

Y-KL conceived the research. JH, R-XZ, DK, PS, HL, and ZL conducted experiments. JH, R-XZ, AH, and Y-KL analyzed data and wrote the manuscript with the support of DK, PS, and ZL. All authors read and approved the manuscript.

## Conflict of Interest

The authors declare that the research was conducted in the absence of any commercial or financial relationships that could be construed as a potential conflict of interest.

## Footnotes

1 https://www.genevestigator.com

2 http://bar.utoronto.ca/eplant/

## Supplementary Material

FIGURE S1 ROS accumulation is disrupted in the *big* mutant during stomatal closure induced by eCO_2_. **(A)** eCO_2_-induced ROS production during eCO_2_-induced stomatal closure is reduced in comparison to WT. Mean H_2_DCF-DA fluorescence intensity was measured on 2.5 h light-preincubated epidermal peels, treated with CO_2_-free (mock) or 800 ppm CO_2_ (elevated CO_2_) for another 2.5 h. **(B)** In the inhibition of light-stimulated stomatal opening by eCO_2_, ROS production in WT and *big* mutants is identical. Mean H_2_DCF-DA fluorescence intensity was measured on light-incubated epidermal peels treated with CO_2_-free (mock) or 800 ppm CO_2_ (elevated CO_2_) for 3 h. In **(A,B)** (*n* = 60), values are means ± s.e. All experiments were repeated at least three times. Different letters indicate significant differences at *P* < 0.05 based on a Tukey’s test.

FIGURE S2 ROS generation related genes transcription levels are induced by eCO_2_. **(A**–**F)** four-week old intact leaves were treated with or without eCO_2_ for 3 h, the transcription levels of *PRX33* **(A)**, *PRX34* **(B)**, *RBOHD* **(C),** and *RBOHF* **(D)** genes were determined by quantitative RT-PCR and normalized to *Actin3*, *OST1* **(E)** and *SLAC1* **(F)** were used as positive controls. In **(A–F)**, the shown result is a representative of three independent biological experiments, values are mean ± s.e. Means with different letters represent statistically significant differences at *P* < 0.05 based on a Tukey’s test.

FIGURE S3 eCO_2_-induced ROS accumulation requires SA signaling. eCO_2_ stimulates an increase H_2_DCFDA fluorescence in WT guard cells, but is blocked in *npr3npr4* mutants. Mean fluorescence intensity was measured on 2.5 h light-preincubated epidermal peels, treated with 800 ppm CO_2_ for another 2.5 h. Values are mean ± s.e. (*n* = 50). All experiments were repeated at least three times. Different letters represent statistically significant differences at *P* < 0.05 based on a Tukey’s test.

FIGURE S4 eCO_2_-induced stomatal closure requires JA signaling. **(A)** eCO_2_-induced stomatal closure is disrupted in *coi1-1* mutants. **(B)** eCO_2_-induced stomatal closure is disrupted in *jar1-1* mutants. Stomatal apertures in **(A,B)** were measured on 2.5 h light-preincubated epidermal peels, treated with 800 ppm CO_2_ for another 2.5 h. In **(A,B)**, the shown result is a representative of three independent biological experiments, values are mean ± s.e. (*n* = 120). Different letters represent statistically significant differences at *P* < 0.05 based on a Tukey’s test.

FIGURE S5 The expression of *PRXs* and *RBOH* gene are not affected in ROS mutants. Four-week old leaves were used to extract mRNA. The quantitative RT-PCR **(A**–**D)** and RT-PCR **(E)** analysis of *PRX33*, *PRX34*, *RBOHD*, and *RBOHF* transcription in leaves of 5-week-old WT, *prx33-3*, *prx34-2*, and *rbohDrbohF* mutants. For quantitative RT-PCR, the transcription levels normalized to *Actin3*; for RT-PCR, *EF1a* was used as a control for cDNA quantity. In **(A–D)**, the shown result is a representative of three independent biological experiments, values are mean ± s.e. Different letters represent statistically significant differences at *P* < 0.05 based on a Tukey’s test.

FIGURE S6 Expression of *RBOHs* genes in leaves and guard cells after treatment with ABA. Heat map showing levels of expression of *AtRBOHA- AtRBOHJ* genes (log_2_ intensity) in guard cells (1–6) and leaves (7–10) according to ePlant (http://bar.utoronto.ca/eplant/). 1 represents the mock test, 2 is treated with 50 μM ABA for 20 h (reference to Böhmer and Schroeder, 2011), 3 and 7 represent mock tests, 4 and 7 are treated with 100 μM ABA for 4 h (reference to Yang et al., 2008), 5 and 9 represent mock tests, 6 and 10 are treated with 50 μM ABA for 3 h (reference to Pandey et al., 2010).

TABLE S1 Mutants used in this study.

TABLE S2 Primers used in this study.

## Abbreviations

ABA: abscisic acid
CA: carbonic anhydrase
CO_2_: carbon dioxide
eCO_2_: elevated carbon dioxide
DPI: diphenylene iodinium
DMSO: N,N-dimethylsphingosine
H_2_O_2_: hydrogen peroxide
H_2_DCF-DA: 2 ′,7 ′ -dichlorodihydrofluorescein diacetate
JA: jasmonic acid
MES: 2-[N]-morpholinnoethane sulfonic acid
NADPH: nicotinamide adenine dinucloetide phosphate
NO: nitric oxide
PAMP: pathogen-associated molecular pattern
PAOs: polyamine oxidases
PCR: polymerase chain reaction
PRXs: peroxidases
RBOH: respiratory burst oxidase
ROS: reactive oxygen species
RT-PCR: reverse transcription-polymerase chain reaction
SHAM: salicylhydroxamic acid
SA: salicylic acid
SAR: systemic acquired resistance
WT: wild type.
