# Supplementary figures and images for: ROS of Distinct Sources and Salicylic Acid Separate Elevated CO2-Mediated Stomatal Movements in Arabidopsis
Source: Front Plant Sci. 2020 May 8;11:542. doi: 10.3389/fpls.2020.00542 (PMC7225777; doi:10.3389/fpls.2020.00542)

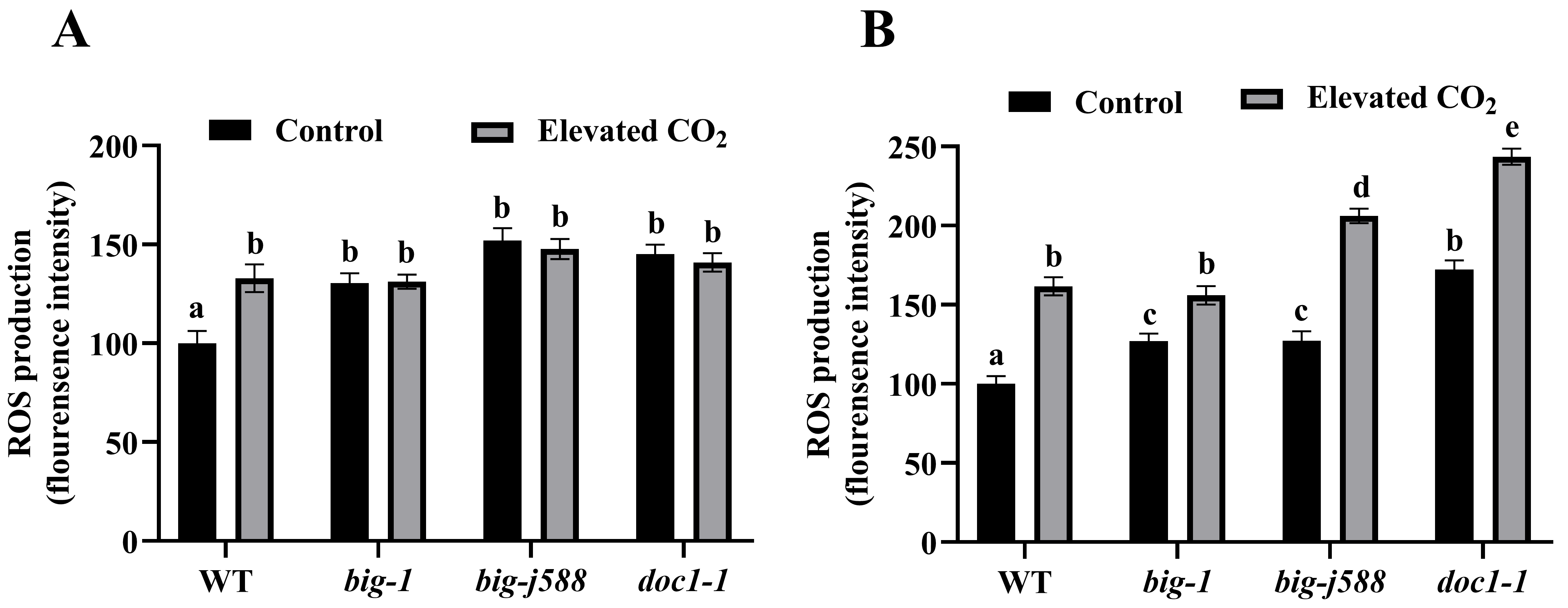

Supplement: FIGURE S1 — ROS accumulation is disrupted in the big mutant during stomatal closure induced by eCO2. (A) eCO2-induced ROS production during eCO2-induced stomatal closure is reduced in comparison to WT. Mean H2DCF-DA fluorescence intensity was measured on 2.5 h light-preincubated epidermal peels, treated with CO2-free (mock) or 800 ppm CO2 (elevated CO2) for another 2.5 h. (B) In the inhibition of light-stimulated stomatal opening by eCO2, ROS production in WT and big mutants is identical. Mean H2DCF-DA fluorescence intensity was measured on light-incubated epidermal peels treated with CO2-free (mock) or 800 ppm CO2 (elevated CO2) for 3 h. In (A,B) (n = 60), values are means ± s.e. All experiments were repeated at least three times. Different letters indicate significant differences at P < 0.05 based on a Tukey’s test. [file Image_1.TIF]

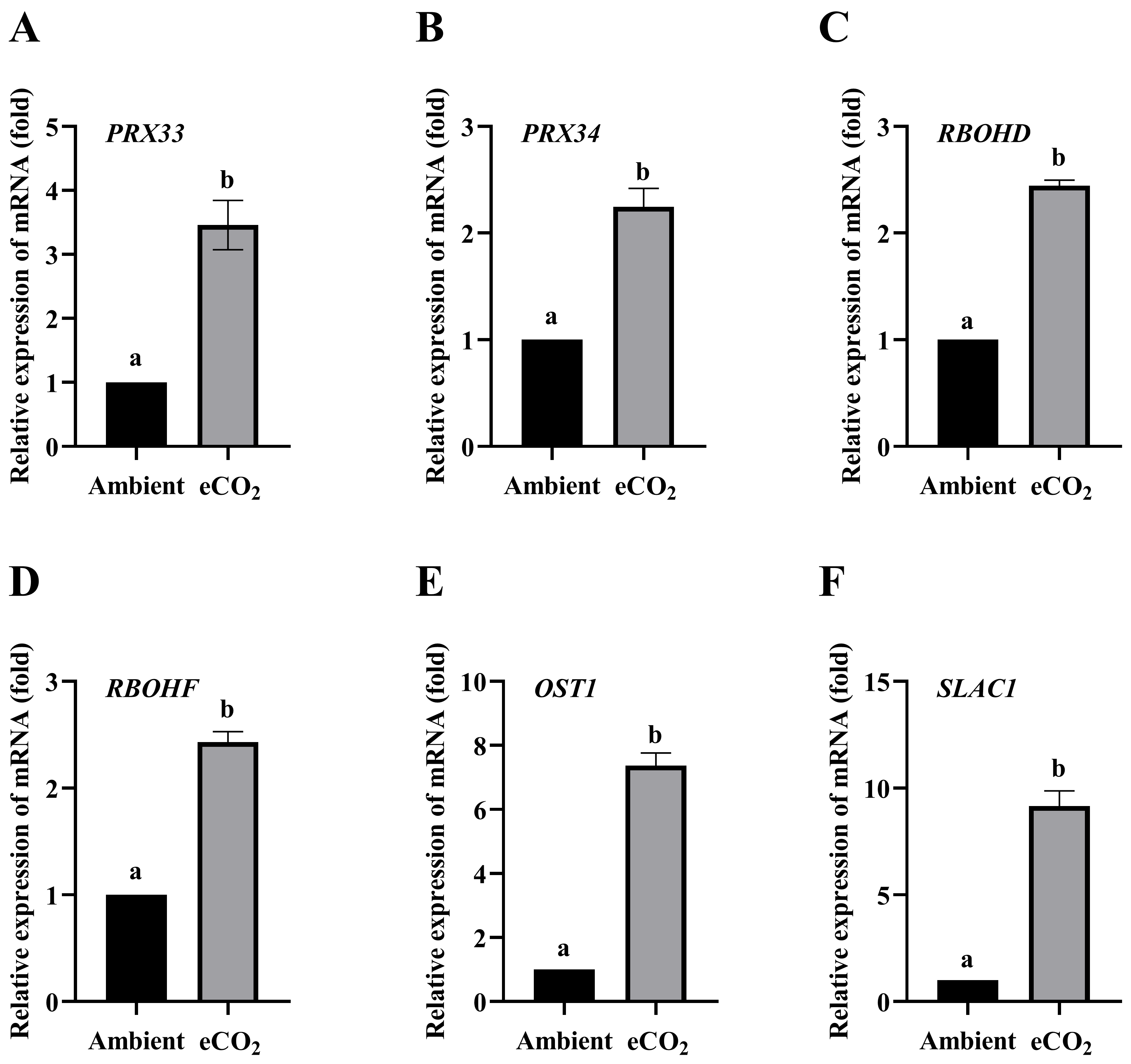

Supplement: FIGURE S2 — ROS generation related genes transcription levels are induced by eCO2. (A–F) four-week old intact leaves were treated with or without eCO2 for 3 h, the transcription levels of PRX33 (A), PRX34 (B), RBOHD (C), and RBOHF (D) genes were determined by quantitative RT-PCR and normalized to Actin3, OST1 (E) and SLAC1 (F) were used as positive controls. In (A–F), the shown result is a representative of three independent biological experiments, values are mean ± s.e. Means with different letters represent statistically significant differences at P < 0.05 based on a Tukey’s test. [file Image_2.TIF]

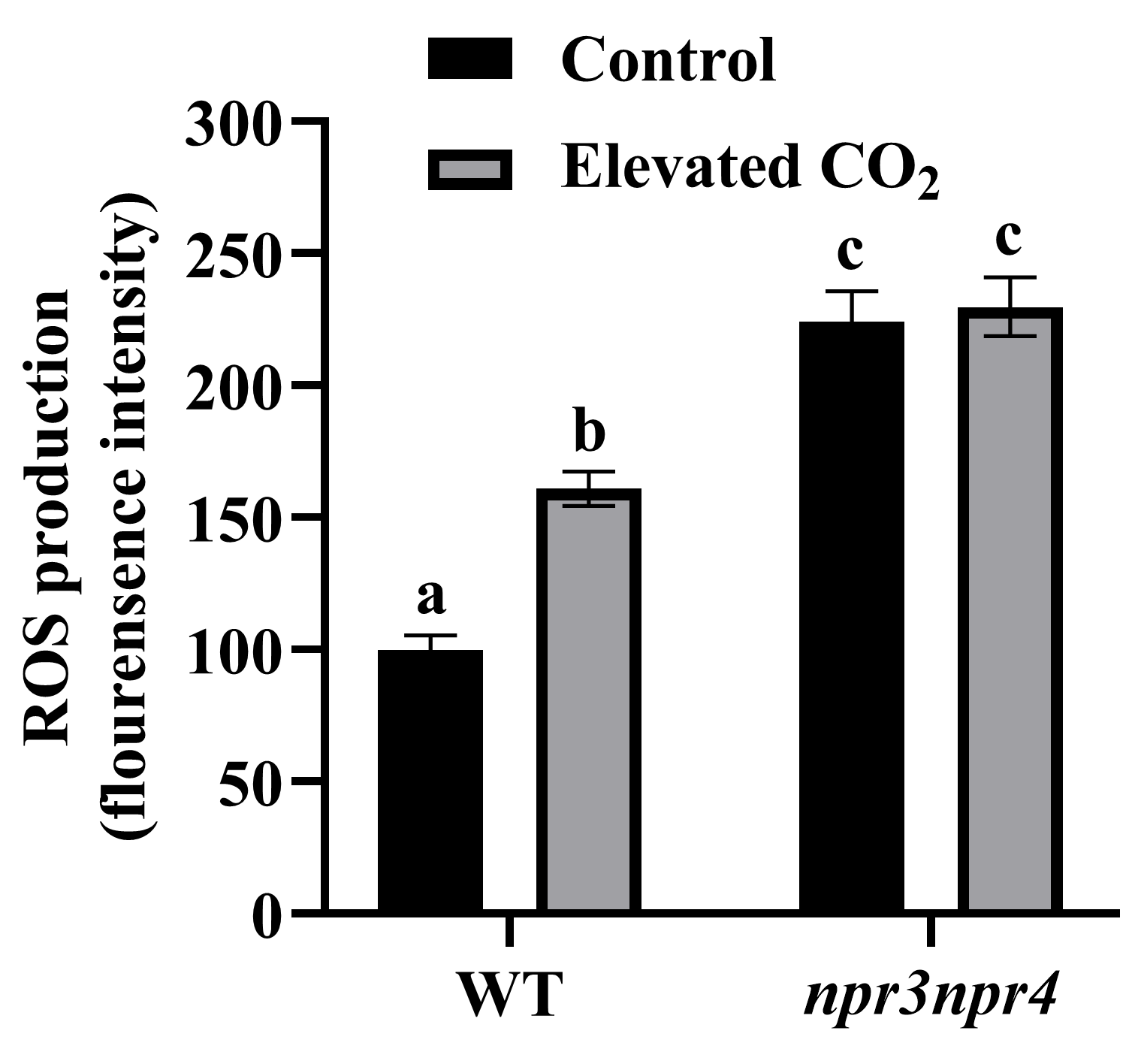

Supplement: FIGURE S3 — eCO2-induced ROS accumulation requires SA signaling. eCO2 stimulates an increase H2DCFDA fluorescence in WT guard cells, but is blocked in npr3npr4 mutants. Mean fluorescence intensity was measured on 2.5 h light-preincubated epidermal peels, treated with 800 ppm CO2 for another 2.5 h. Values are mean ± s.e. (n = 50). All experiments were repeated at least three times. Different letters represent statistically significant differences at P < 0.05 based on a Tukey’s test. [file Image_3.TIF]

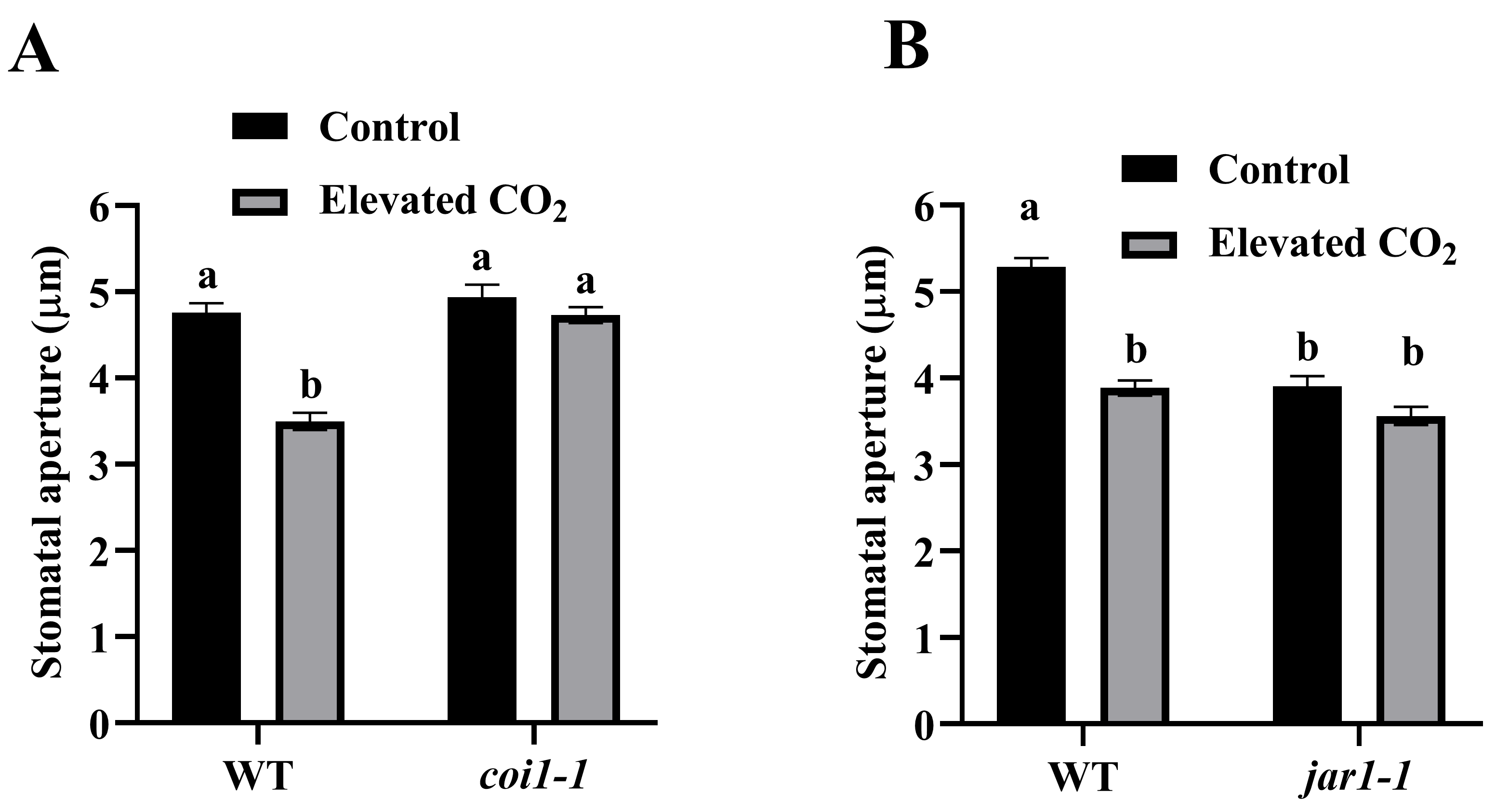

Supplement: FIGURE S4 — eCO2-induced stomatal closure requires JA signaling. (A) eCO2-induced stomatal closure is disrupted in coi1-1 mutants. (B) eCO2-induced stomatal closure is disrupted in jar1-1 mutants. Stomatal apertures in (A,B) were measured on 2.5 h light-preincubated epidermal peels, treated with 800 ppm CO2 for another 2.5 h. In (A,B), the shown result is a representative of three independent biological experiments, values are mean ± s.e. (n = 120). Different letters represent statistically significant differences at P < 0.05 based on a Tukey’s test. [file Image_4.TIF]

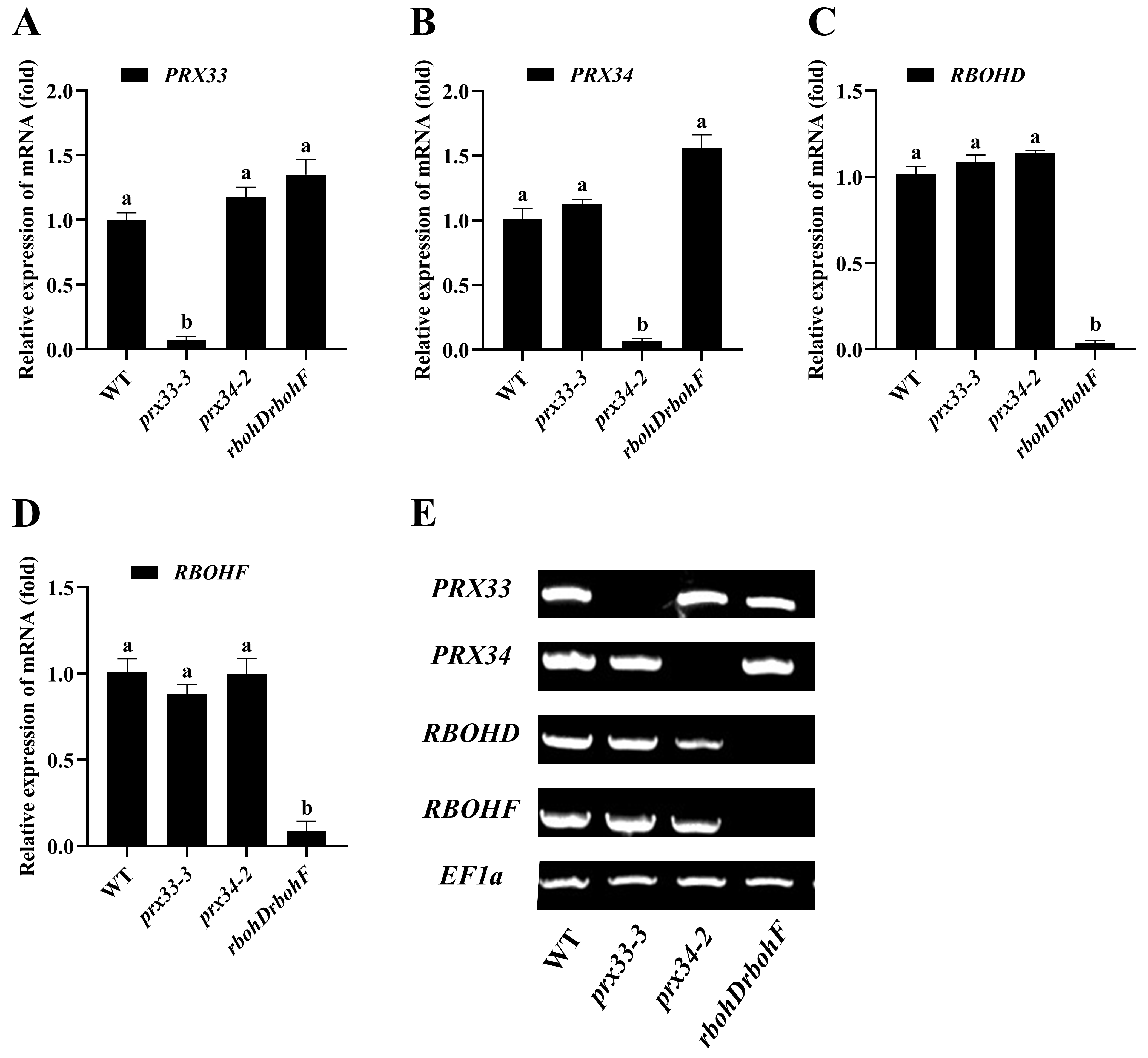

Supplement: FIGURE S5 — The expression of PRXs and RBOH gene are not affected in ROS mutants. Four-week old leaves were used to extract mRNA. The quantitative RT-PCR (A–D) and RT-PCR (E) analysis of PRX33, PRX34, RBOHD, and RBOHF transcription in leaves of 5-week-old WT, prx33-3, prx34-2, and rbohDrbohF mutants. For quantitative RT-PCR, the transcription levels normalized to Actin3; for RT-PCR, EF1a was used as a control for cDNA quantity. In (A–D), the shown result is a representative of three independent biological experiments, values are mean ± s.e. Different letters represent statistically significant differences at P < 0.05 based on a Tukey’s test. [file Image_5.TIF]

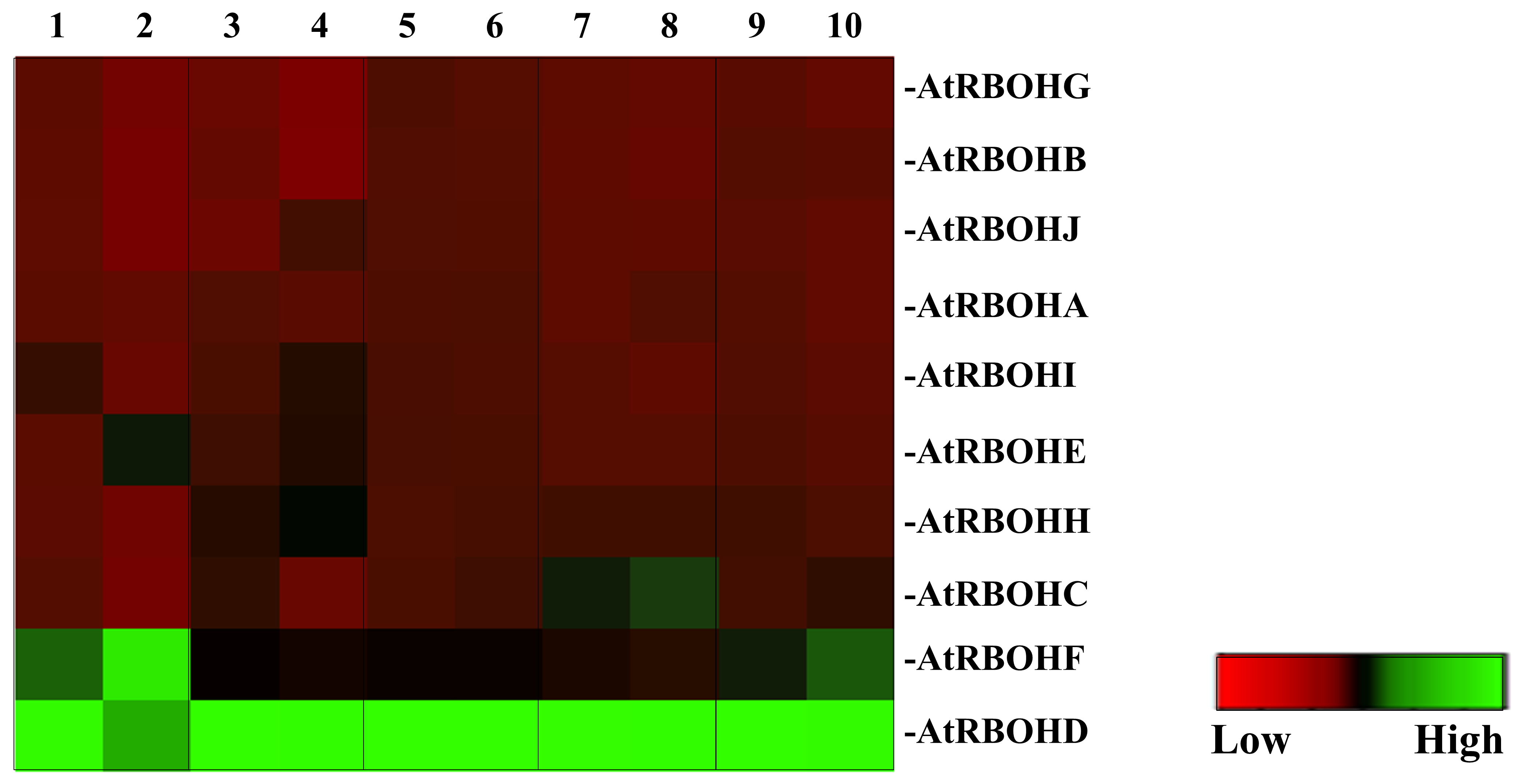

Supplement: FIGURE S6 — Expression of RBOHs genes in leaves and guard cells after treatment with ABA. Heat map showing levels of expression of AtRBOHA- AtRBOHJ genes (log2 intensity) in guard cells (1–6) and leaves (7–10) according to ePlant (http://bar.utoronto.ca/eplant/). 1 represents the mock test, 2 is treated with 50 μM ABA for 20 h (reference to Böhmer and Schroeder, 2011), 3 and 7 represent mock tests, 4 and 7 are treated with 100 μM ABA for 4 h (reference to Yang et al., 2008), 5 and 9 represent mock tests, 6 and 10 are treated with 50 μM ABA for 3 h (reference to Pandey et al., 2010). [file Image_6.TIF]
