# Supplementary material for: ROS of Distinct Sources and Salicylic Acid Separate Elevated CO2-Mediated Stomatal Movements in Arabidopsis
Source: Front Plant Sci. 2020 May 8;11:542. doi: 10.3389/fpls.2020.00542 (PMC7225777; doi:10.3389/fpls.2020.00542)
Supplement: TABLE S1 — Mutants used in this study. [file Table_1.DOCX]

**Supplemental Table 1. Mutants used in this study.**

| **Mutants** | **Function** |
| --- | --- |
| *sid2-2* | Disrupt SA biosynthesis |
| *npr1-1* | Disrupt SA signaling transduction |
| *npr3npr4* | SA receptor mutants: disrupt SA signaling transduction |
| *prx33-3* | Decrease peroxidase mediated ROS generation |
| *prx34-2* | Decrease peroxidase mediated ROS generation |
| *rbohDrbohF* | Decrease NADPH oxidases mediated ROS generation |
| *ost1-3* | Loss response to ABA-induced stomatal closure |
| *ABA1124* | ABA receptor mutants |
| *coi1-1* | JA pathway deficient mutant |
| *myc2-2* | Disrupt JA signaling transduction |
| *jar1-1* | JA pathway deficient mutant |
| *big-1* | *BIG* gene allele mutant |
| *big-j588* | *BIG* gene allele mutant |
| *doc1-1* | *BIG* gene allele mutant |
