# Supplementary material for: ROS of Distinct Sources and Salicylic Acid Separate Elevated CO2-Mediated Stomatal Movements in Arabidopsis
Source: Front Plant Sci. 2020 May 8;11:542. doi: 10.3389/fpls.2020.00542 (PMC7225777; doi:10.3389/fpls.2020.00542)
Supplement: TABLE S2 — Primers used in this study. [file Table_2.DOCX]

**Supplemental Table 2. Primers used in this study.**

| **Genes** | **primers 5’ to 3’** |
| --- | --- |
| **Quantitative RT-PCR** |  |
| ACT3 (F) | GGCAGAATATGATGAGTCAGG |
| ACT3 (R) | AAAGAAGAGCAGAGAACGAAG |
| RBOHD (F) | TCCACGTTTAATTGCCGC |
| QRBOHD (R) | TCCATCCTTCCACTCCTTTC |
| QRBOHF (F) | ACAGCGCGTGAAAAATGG |
| QRBOHF (R) | GAAAGAGAGAGAGAGAGGGAG |
| QPRX33 (F) | ATCGTCCTTCTGATCTTGTTGCG |
| QPRX33 (R) | GCAGATCGAAATCCACTAAGACG |
| QPRX34-(F) | ATCGTCCTTCTGATCTCGTTGCT |
| QPRX34 (R) | GATCAAAATCTACCAAGGCACTTC |
| **RT-PCR** |  |
| EF1*ɑ* (F) | ATGCCCCAGGACATCGTGATTTCAT |
| EF1*ɑ* (R) | TTGGCGGCACCCTTAGCTGGATCA |
| PRX33 (F) | ACTTACCCCTACTTTTTACGACACT |
| PRX33 (R) | GCAGATCGAAATCCACTAAGACG |
| PRX34 (F) | TCACCCCTACCTTCTACGATAG |
| PRX34 (R) | GATCAAAATCTACCAAGGCACTTC |
| RBOHD (F) | CCGACGATCTTCCCAAGAGA |
| RBOHD (R) | CCTGGCATTCCACAGTAG |
| RBOHF (F) | ACCGCCATTAATGTCATCGG |
| RBOHF (R) | GCATTGCAATGCTTGGAAC |
| **Genotyping** |  |
| PRX33-3 (F) | TGGAAATGCAAATTCAGCCCGA |
| PRX33-3 (R) | CAGATCGAAATCCACTAAGACG |
| GABI-Kat T-DNA | ATATTGACCATCATACTCATTGC |
| PRX34-2 (F) | ACAGCGCGTGAAAAATGG |
| PRX34-2 (R) | GAAAGAGAGAGAGAGAGGGAG |
| RbohD (F) | GATGGGAGACAGCAGGAT |
| RbohD (R) | CGGATATGTACGCTCAGGT |
| dSpm1 | CTTATTTCAGTAAGAGTGTGGGGTTTTGG |
| RbohF (F) | CTTCCGATATCCTTCAACCAACTC |
| RbohF (R) | TCGGAGCGATAGATGTAACCAT |
